# The limits of the foreign language effect on decision-making: The case of the outcome bias and the representativeness heuristic

**DOI:** 10.1371/journal.pone.0203528

**Published:** 2018-09-07

**Authors:** Marc-Lluís Vives, Melina Aparici, Albert Costa

**Affiliations:** 1 Center for Brain and Cognition, Universitat Pompeu Fabra, Barcelona, Spain; 2 Departament de Psicologia Bàsica, Evolutiva i de l'Educació, Universitat Autònoma de Barcelona, Barcelona, Spain; 3 Institució Catalana de Recerca i Estudis Avançats (ICREA), Barcelona, Spain; Universidad Torcuato Di Tella, ARGENTINA

## Abstract

Language context (native vs. foreign) affects people’s choices and preferences in a wide variety of situations. However, emotional reactions are a key component driving people’s choices in those situations. In six studies, we test whether foreign language context modifies biases and the use of heuristics not directly caused by emotional reactions. We fail to find evidence that foreign language context modifies the extent to which people suffer from outcome bias (Experiment 1a & 1b) and the use of the representativeness heuristic (Experiment 2a & 2b). Furthermore, foreign language context does not modulate decision-making in those scenarios even when emotion is brought into the context (Experiment 1c & 2c). Foreign language context shapes decision-making, but the scope of its effects might be limited to decision-making tendencies in which emotion plays a causal role.

## Introduction

The linguistic context (native vs. foreign language) in which people are immersed in is one of those supposed irrelevant factors [1] that affect people’s decisions, choices and revealed preferences [2,3]. Presenting problems in people’s foreign language seems to promote a more deliberative thinking style that results in a reduction of certain decision biases at least in some decision-making contexts—the so-called foreign language effect on decision making (FLE). For example, foreign language contexts reduce framing effects and the aversion to ambiguity, risk and losses [4–6]. Furthermore, it also reduces the hot-hand fallacy, magical thinking, and the illusion of causality [7–9].

A proper understanding of the contexts in which foreign language may affect our revealed preferences is important given that many people must make decisions in such contexts. Think of the large amount of people that are making political and business decisions in contexts other than their native language. However, our current knowledge of the pervasiveness of this effect is rather limited, and hence before one is tempted to propose the use of a foreign language as a “nudge” to improve certain decisions, we need to study which contexts are sensitive to this effect [10,11]. We approach this goal by assessing the effect of foreign language context in three different decision situations that lead to well-known biases: the outcome bias, the conjunction and base-rate neglect fallacies [12–14]. To advance the results: people’s decisions in these situations do not seem to be affected by the linguistic context.

### Foreign language effect: A reduction in emotion or an increase in deliberation?

Two main non-mutually exclusive mechanisms for the FLE have been proposed. According to the *reduced emotionality account* [15,16], a FL context reduces the emotional reactivity that certain scenarios may elicit [17–21]. This reduction in emotional reactivity may then impact people’s decisions, sometimes reducing biases that are related with such emotional processing (e.g., loss aversion). In contrast, according to *the cognitive enhancement hypothesis*, the FLE comes about because an increased in analytic (or deliberative) processing, promoted by a foreign language context [5,16,22]. This increase in deliberation may come about because the cognitive burden that FL processing poses, which then may prompt people to be more careful (and slower) when assessing the options afforded by the problem. This, in turn, may help to overcome the prominent intuitive responses that sometimes these problems elicit, hence causing people to rely less on intuition and perhaps more on deliberation. As we have put it elsewhere, foreign language contexts may help you to think twice when confronted with difficult problems [5].

These two different mechanisms make different predictions regarding the scope of FLEs in decision-making: while the *emotionality account* posits that a foreign language effect will only (or mostly) be present when emotion plays a major role in the decision process, the *cognitive enhancement account* predicts that the effect will be present in a wider set of contexts that elicit intuitive responses.

FLEs have been mostly explored in decision making scenarios involving some sort of emotional connotation. Hence, it is unclear whether such an effect will be circumscribed to such emotional contexts, as the reduced emotionality account would predict, or rather it would extend to more emotionally-neutral ones as suggested by the cognitive enhancement hypotheses. In fact, attempts to disentangle both of these accounts have been pursued in the context of the FLE in moral decisions. According to these studies, foreign language effect in moral judgments would respond more to a reduction in the emotional intensity with which the scenario is perceived and not to an increase in deliberation per se [23]. This is one of the few evidences that we have suggesting that a reduced in emotional reactivity is the force behind the FLE.

### The current study: Outcome bias and the representativeness heuristic

We assess the presence of a FLE in three well-known biases: the outcome bias, and the conjunction and base-rate neglect fallacies.

The outcome bias refers to people’s tendency to evaluate the goodness of a decision by assessing its outcome: we tend to judge decisions as more appropriate if they have resulted in a desirable outcomes than if they have not [12]. This is considered a bias, since when one is making the decision its outcome is not known and, consequently, should not be factored in when evaluating the goodness of the decision.

The conjunction and base-rate neglect fallacies are related to the representative heuristic: people’s tendency to make probability judgments relying on how similar a given prospect is to the target population while neglecting other information that is (or might be) more informative [24]. The conjunction fallacy refers to judging the presence of two events (A&B) as more probable than one of its constituents (A; B) [13]. The base-rate neglect fallacy refers to the tendency to make probability judgments without considering the base-rate in the population [14,25].

We chose to explore presence of a FLE in these three effects because:

- They arguably involve a relatively low emotional impact, and therefore can help to contrast the two hypotheses mentioned above.
- They are quite prevalent and easy to elicit. This gives room for detecting the potential modulation that linguistic context may exert.
- Previous work suggests that foreign language use might have an effect on these contexts [7,15,26].

## Results

### Experiments 1a-1c: Does foreign language context reduce the outcome bias?

In Experiments 1a and 1b we use two scenarios (see below) that were identical in their introduction but had different outcomes (positive or negative) [12]:

Common Introduction: A 55-year-old man had a heart disease. He had to stop working because of the pain it caused him. He enjoyed his work and did not want to stop […]. A heart operation would reduce his pain and increase his life expectancy from age 65 to age 70. However, 8% of the people who have this operation die from the operation itself. His doctor decided to do the operation

Positive Outcome: *The operation went well*, *and the man recovered*.

Negative Outcome: *The operation failed*, *and the man died*.

We decided to conduct two experiments: one in which participants saw the two scenarios (within-subjects; one with positive and one with negative outcomes) and one in which participants saw only one scenario (between-subjects: with positive or negative outcome). This was done because both designs have their own virtues. On one hand, within-subject designs are more powerful and guarantee that the individual variability is controlled for both positive and negative outcomes. However, such design may lead to a reduction in outcome bias, given the carry over effects that responding to one scenario may have on the subsequent response to the other scenario. That is, people may try to be more consistent in their evaluations reducing the impact of the outcome on their decisions. This problem is solved by using between-subject design, at the expenses however of reducing power.

In Experiment 1c, we use a different scenario in which participants were asked to evaluate the goodness of their own decisions, rather than evaluating other people’s decisions in the financial domain. We make this modification with the objective to increase the variety of situations in which we test the foreign language effect, thus increasing the generalizability of our findings.

#### Method

Participants were tested in classrooms (of about 50 people) from several universities in Barcelona, Spain (except for Experiment 1c and 2c, see detailed Methods below). They were all native or near native Spanish speakers–acquired the language before 4 years of age and use it in their daily basis. Most participants had English as a foreign language, a language acquired in a classroom setting. Those participants who lived more than 12 months in an English-speaking country were discarded.

Participants were presented with the problems either in their foreign (English) or native language (Spanish), hence the language factor was always a between participant factor. The whole experimental session was carried out in the same language (either Spanish or English), with a high proficient Spanish-English speaker as an experimenter. Language condition was assigned randomly to each classroom. After rating the scenarios, participants filled an English self-reported proficiency questionnaire, and were asked to rate their understanding of the scenario. Participants who reported understanding less than 50% of the problem were excluded (less than 1%). This procedure was followed in all the experiments.

One hundred seventy-eight Spanish native speakers participated in Experiment 1a. Two were removed because they lived more than 12 months in an English-speaking country, resulting in a final N = 176 (N for native language (NL) = 88, N for foreign language (FL) = 88; Gender NL = 66% women (W); Gender FL = 76% W; Age NL = 19; Age FL = 19).

Seven hundred and eleven students participated in Experiment 1b. Seventeen were excluded because they were not native or near native Spanish speakers, seven because they lived more than 12 months in an English-speaking country, and three because they reported to understand less than 50% of the English language. This led to a final N = 684 (N_NL_ = 346, N_FL_ = 338; Gender NL = 63% W; Gender FL = 65% W; Age NL = 21; Age FL = 21).

Two different scenarios (translated from English into Spanish and back-translated into English to guarantee language equivalence [27]) were used as materials (see above). Participants were asked to evaluate the decision in a scale ranging from 1 (very bad decision) to 7 (excellent decision). In Experiment 1a participants saw both scenarios (with order counterbalanced) while in Experiment 1b they only saw one. After rating the scenarios, participants filled an English self-reported proficiency questionnaire, and were asked to rate their understanding of the scenario. Participants who reported understanding less than 50% of the problem were excluded (less than 1%). Thus, all participants had a moderate level of English (see Table 1 for English level descriptive statistics for all the experiments).

**Table 1:** Descriptive statistics of foreign language (English) proficiency.

Experiment	Reading	Writing	Listening	Speaking
Exp 1a	5.65	4.97	5.81	4.79
Exp 1b	5.70	4.90	5.68	4.78
Exp 1c	5.92	5.02	5.92	5.02
Exp 2a	5.56	4.86	5.63	4.75
Exp 2b	5.60	4.50	5.40	4.29
Exp 2c	5.87	4.96	5.91	5.04

Self-rated English proficiency (1 = low proficiency, 7 = high proficiency) for Reading, Writing, Listening, and Speaking broken down by Experiment.

The whole experimental session was carried out in the same language (either Spanish or English), with a high proficient Spanish-English speaker as an experimenter. Language condition was assigned randomly to each classroom. This research and all the following were approved by the Clinical Research Ethical Committee at Pompeu Fabra University. Participants were given written or oral informed consent in all the experiments.

#### Results

Experiment 1a (within-subjects design): Decisions that led to a positive outcome were better rated than those leading to a negative one: *F*(1,174) = 45.45, *p* < 0.001, which reveals the presence of an outcome bias (see Table 2). No differences in overall ratings were observed across languages: *F*(1,174) = 0.64, *p* = 0.42. Importantly, the magnitude of the outcome bias was similar in both language contexts as revealed by the lack interaction between outcome and language: *F*(1,174) = 0.98, *p* = 0.32.

**Table 2:** Results Experiments 1a and 1b.

	Experiment 1a			Experiment 1b		
	Outcome			Outcome		
	Positive	Negative	Δ P-N	Positive	Negative	Δ P-N
Native Language	5.66 (*0*.*80*)	5.29 (*1*.*00*)	0.37***	5.54 (*1*.*08*)	5.12 (*1*.*14*)	0.42***
Foreign Language	5.61 (*0*.*96*)	5.13 (*1*.*16*)	0.48***	5.44 (*1*.*07*)	4.90 (*1*.*21*)	0.54***
Difference	0.05	0.16	-0.11	0.10	0.22	-0.12

Average evaluations of the doctor’s decision (1 (very bad) to 7 (very good)) broken by outcome, language and Experiment (standard deviations in parenthesis).

*** p < 0.001.

Experiment 1b (between-subjects design): Decisions that led to a positive outcome were evaluated better than decisions that led to a negative one: *F*(1,680) = 31.52, *p* < 0.001 (see Table 2), and ratings were similar in the two languages *F*(1,680) = 3.66, *p* = 0.06. Again, the interaction between type of outcome and language context was not significant: *F*(1,680) = 0.64, *p* = 0.42.

These results show no effect of foreign language context on people’s outcome bias. In Experiment 1c, we further explore this issue in a context that increases participant’s involvement in the decision process [28].

### Experiment 1c: The outcome bias in personal decision-making

Here we investigated the foreign language effect on the outcome bias in a new scenario. This was done to increase the scope in which we explored the outcome bias. This time, the scenario was related to the financial domain, not the health one. Furthermore, we asked participants to evaluate the goodness of a decision that has been previously taken by themselves in a hypothetical context. Hence, unlike Experiments 1a and 1b, here we ask participants to judge their own decisions after knowing the corresponding outcomes instead of evaluating a decision made by someone else.

#### Method

One hundred eighty-nine participants were tested. Using the previous exclusion criteria, we excluded 32 participants, resulting in a final of N = 159 (N_NL_ = 81, N_FL_ = 78; Gender NL = 65% W; Gender FL = 70% W; Age NL = 22; Age FL = 21).

Participants were tested in the laboratory in groups of 20 in separate individualized computers. The total session lasted about 20 minutes and they were paid 5€ for their participation. They were presented with the following scenario [28]:

Imagine that you want to invest 5,000€ and you must choose between one of two brokers, Broker A and Broker B. A successful investment would mean to increase your 5000€ investment by 15% or more within a year. Broker A has a 43% chance of success, while Broker B has a 54% chance. Which Broker would you choose?

After choosing the broker, participants responded to various questions unrelated to the scenario and to the current study for a period of 20 minutes, always in the same language (either Native or Foreign). Then they were informed about the outcome of their decision. There were two potential outcomes: a) positive outcome (their decision led to a 750€ gain from their initials 5,000€), b) negative outcome (their decision led to a 750€ loss from their initials 5,000€). Participants were then asked to rate (scale from 1 to 7 not at all, very much) the following questions:

- *How much do you regret your decision*?
- *How good do you think your decision was*?
- *How much do you wish to have chosen the other broker*?
- *Which broker would you choose in case of doing a new investment*?

After responding these questions, and to guarantee comparable understanding of the problem in both language contexts, we asked participants to recall broker’s success chances.

#### Results

Decisions that resulted in a negative outcome led to different ratings as compared those that resulted in a positive outcome. They led to feel more regret about the taken decision (*F*(1,155) = 104.76, *p* < 0.001), to think that the decision was worse (*F*(1,155) = 57.76, *p* < 0.001); to wish to have chosen the other option (*F*(1,155) = 48.90, *p* < 0.001) [28] (see Table 3). Participants also reported that they would change their choice if they were to make a new investment more often in the negative outcome than in the positive one: *χ*^2^(1, N = 159) = 17.27, p < 0.001.

**Table 3:** Results Experiment 1c.

	Regret			Wish			Goodness		
	Outcome			Outcome			Outcome		
	Pos.	Neg.	Δ P-N	Pos.	Neg.	Δ P-N	Pos.	Neg.	Δ P-N
Native Language	1.23 (*0*.*48*)	3.56 (*1*.*82*)	-2.33***	1.26 (*0*.*53*)	2.95 (*1*.*96*)	-1.69***	6.28 (*1*.*40*)	4.93 (*1*.*71*)	1.35***
Foreign Language	1.55 (*1*.*25*)	3.85 (*1*.*70*)	-2.30***	1.66 (*1*.*22*)	3.36 (*1*.*91*)	-1.70***	6.36 (*0*.*93*)	3.92 (*1*.*96*)	2.44***
Difference	-0.32	-0.29	0.03	-0.40	-0.41	-0.01	-0.08	1.01	-1.09

Averaged participants’ ratings for how much they regret their decision (Regret), they wished to have chosen the other option (Wish) and how good they thought their decision was (Goodness) on a scale from 1 (not at all) to 7 (very much) broken down by language and outcome (standard deviations in parenthesis).

*** p < 0.001.

Language did not affect these ratings (regret: *F(*1,155) = 1.91, *p* = 0.16; changing decision *F(*1,155) = 3.00, *p* = 0.08; or goodness of the decision *F(*1,155) = 3.36, *p* = 0.06). However, the last two approach significance, suggesting that people might have used the scale differently in their foreign language (changing decision: *M*_*Native*_ = 2.10, *SD*_*Native*_ = 1.68; *M*_*Foreign*_ = 2.58, *SD*_*Foreign*_ = 1.83; goodness of the decision: *M*_*Native*_ = 2.41, *SD*_*Native*_ = 1.69; *M*_*Foreign*_ = 2.95, *SD*_*Foreign*_ = 1.98)

Importantly, no interaction between language and outcome was found for the questions about the regret and the wish to have chosen the other broker: *F(*1,155) = 0.005, *p* = 0.94; *F(*1,155) = 0.005, *p* = 0.94, respectively. However, a significant interaction was found when evaluating the goodness of the decision: *F(*1,155) = 4.75, *p* = 0.03, which does not survive to Bonferroni-corrections for multiple comparisons (in this case, we applied it because we used four different dependent variables). Finally, the intention to change brokers was not affected language context: *χ*^2^(1, N = 159) = 0.25, p = 0.97 (see Table 3).

To assess memory scores, we created an index of recall by subtracting the correct answer to participants’ responses. Then, we added together the absolute value of Broker A and B’s subtractions. Thus, 0 means perfect recall and any departure from 0 represents the distance from participants’ response to the correct answer. Participants were similarly accurate in native and foreign language: *F(*1,157) = 1.25 *p* = 0.26; *M*_*Native*_ = 15.97, *SD*_*Native*_ = 21.88; *M*_*Foreign*_ = 12.52, *SD*_*Foreign*_ = 16.57). Foreign language use does not impair memory recollection.

The results of Experiments 1a-1c show that language context does not affect the magnitude of the outcome bias. Hence, we fail to find evidence that language context alters the weight given to the outcome when evaluating the appropriateness of a decision.

### Experiments 2a-2c: Does foreign language context reduce the use of the representativeness heuristic?

We explore how language context affects another major factor driving people’s decisions: the representative heuristic. If a foreign language context promotes a more careful and deliberative thinking, then it is possible that it helps people to treat probabilities in a more normative way. If so, and to the extent that the representative heuristic is driven by stereotypical information against the evaluation of actual probabilities, one would expect a reduction of its application in a foreign language context. We put to test this hypothesis by assessing the conjunction fallacy and the base-rate neglect fallacy that index the application of such heuristic.

### Experiment 2a: Does foreign language context reduce the conjunction fallacy?

The conjunction fallacy refers to judging the presence of two events (A&B) as more probable than one of its constituents (A; B), which is logically faulty. This fallacy seems to stem from participants guiding their responses paying more attention to the stereotypical information provided by the scenario than from the actual probabilities provided.

#### Method

Five hundred sixteen participants were tested following the same procedure as in the previous studies. Using the previous exclusion criteria, we excluded 20 participants, resulting in a final of N = 496 (N_NL_ = 258, N_FL_ = 238; Gender NL = 73% W; Gender FL = 76% W; Age NL = 20; Age FL = 19).

Participants responded to two scenarios. The first scenario was based on the classical Linda problem [13]:

*Linda is 31 years old, single and very smart. She has a degree in philosophy. When she was a student, Linda was concerned about issues of discrimination and social justice, and she participated in anti-globalization protests. Order the following statements according to their probability from most to least probable*.

- *Linda works in a bank*.
- *Linda is an activist in the feminist movement*.
- *Linda works in a bank and is an activist in the feminist movement*.

The conjunction fallacy is considered as any response that orders the conjoint of the two features as more likely as the two features alone.

In the second scenario participants were asked to evaluate which of the following two prospects was most likely to be present in a sample of 1000 people randomly chosen from their city-Barcelona:

- Somebody who suffered a heart attack or
- Somebody who suffered a heart attack and was more than 55 years old.

#### Results

A logistic regression on accuracy as dependent variable (1 = correct, 0 = incorrect) and scenario (Linda or Heart disease) and Language condition (Native or Foreign) as regressors (categorical variables) was performed. Accuracy was higher in the Heart disease item: *B* = 1.24, *z* = 6.01, *p* < 0.001. However, language context did not have any effect on accuracy in any scenario: *B* = -0.15, *z* = -0.67, *p* = 0.50, neither interacted with type of scenario: *B* = 0.33, *z* = 1.14, *p* = 0.25. This shows that participants suffered from the conjunction fallacy to the same degree no matter the language context (see Table 4), suggesting that they apply the representativeness heuristics equally frequently.

**Table 4:** Results Experiments 2a and 2b.

	Experiment 2a		Experiment 2b		
	Conjuction fallacy		Base-rate neglect fallacy		
	Linda problem	Heart disease	Congruent	Neutral	Incongruent
Native Language	18.68%	53.32%	89.65%	72.03%	19.92%
Foreign Language	21.10%	47.90%	93.91%	71.30%	15.22%
Difference	-2.42%	5.42%	-4.26%	0.73%	4.70%

Subjects’ accuracy according to normative logic broken down by type of scenario, language, and Experiment.

### Experiment 2b: Does foreign language context reduce the base-rate neglect fallacy?

We further assess the potential effect of language on the applicability of the representativeness heuristic by means of exploring another fallacy: the base-rate neglect fallacy, which refers to the tendency to make probability judgments without considering the base-rate in the population.

#### Method

Following the same procedure as in the previous studies 504 participants were tested. Using the previous exclusion criteria, we excluded 13 participants, resulting in a final N = 491 (N_NL_ = 261, N_FL_ = 230; Gender NL = 74% W; Gender FL = 74% W; Age NL = 22; Age FL = 22).

Participants were presented with 3 different scenarios [29]. In these scenarios two critical information were provided: a) description of a given person according to a stereotypical profession (e.g., lawyer) and b) the likelihood that someone is an actual lawyer in the population from where the person has been selected:

100 participants participated in this study. 90% were lawyers and 10% were engineers. Jack was one of the participants of the study. Jack is 36 years old. He is not married and is somewhat introverted. He likes to spend his free time reading science fiction and writing computer programs. What is most likely?

- Jack is a lawyer
- Jack is an engineer

We used three items that diverged in the relationship between the base-rate information and the description provided: one was Congruent (the base-rate and the description both pointed to the same response), one was Neutral (the description did not point to any response), and one was Incongruent (the base-rate pointed to one response and the description pointed to the other one, like in the example above). This allowed us to see whether people weighted differently the base-rate and stereotypical information when using their native or foreign language. Participants were presented with all three items and order was counterbalanced across them.

#### Results

Participants’ accuracy (1 = response following base-rate, 0 = response against base-rate) was submitted to a logistic regression with type of item (Neutral, Congruent or Incongruent), Language (Native or Foreign), and the interaction as regressors. As compared to the neutral condition, participants’ accuracy in the congruent item was larger and in the incongruent item was smaller.: *B* = 1.83, *z* = 5.85, *p* < 0.001; *B* = -2.63, *z* = -11.21, *p* < 0.001, respectively (see Table 4). Language context did not change the extent to which participants used the representativeness heuristic as shown by the lack of a main effect of foreign language: *B* = -0.04, *z* = -0.18, *p* > 0.7. Neither modulated the effect of congruency or incongruency: *B* = 0.61, *z* = 1.54, *p* > 0.1; *B* = -0.29, *z* = 0.93, *p* > 0.3, respectively (see Table 4).

The results of Experiments 2a and 2b reveal no evidence that language context affects people’s tendency to use the representativeness heuristic. In other words, people seem to be equally likely to rely on tangential information when evaluating the probability of different events in native and foreign languages.

### Experiment 2c: Does foreign language context reduce the base-rate neglect fallacy with emotional content?

We further explore whether foreign language context may affect the likelihood of with which people apply the representativeness heuristic. However, we do so by increasing the emotionality of the scenario. In particular, we contrast two scenarios that elicit the base-rate neglect fallacy but that diverge in their emotional context. This will allow us to assess whether an emotional context may lead to the appearance of a foreign language effect on base-rate neglect fallacy. Previous research has shown that emotional content causes people to rely more on heuristics and give less weight to base-rates [30]. We tested whether this effect will be reduced when using a foreign language.

#### Method

One hundred eighty-nine participants were tested in the laboratory in groups of 20 in separate individualized computers. The total session lasted about 30 minutes and they were paid 5€ for their participation. Using the previous exclusion criteria, we excluded 13 participants, resulting in a final N = 176 (N_NL_ = 85, N_FL_ = 91; Gender NL = 64% W; Gender FL = 70% W; Age NL = 22; Age FL = 21).

We used similar items than in Experiment 2b but adding emotional content [30]. This time the base rates were different (1000 people, 5 sharing one feature and 995 sharing the other) and the information was either Congruent or Incongruent with the base-rates. In the emotional items, participants had to judge whether somebody from a target population had cancer or anorexia, while in the neutral ones they had to do the same with a medical/lawyer student, or a professional athlete/doctor. Each participant was presented with four items, two emotional (one Congruent, one Incongruent) and two neutral (one Congruent, one Incongruent).

After reading each item, participants were asked to choose which option was most likely and then to report the likelihood that their answer was correct from 0 (not at all likely) to 100 (very likely).

#### Results

We analyzed participants’ estimation of how probable their answer was the correct one [30]. The sign of the probabilities was inversed if the response was against the base-rate, while it was kept the same if it was in concordance with the base-rate.

Congruency had a significant impact in participants’ probability estimates of correctness such as they were more calibrated (followed the base-rate) in the Congruent than the Incongruent condition: *F(*1,174) = 839.45, *p* < 0.001 (see Table 5). This was modulated by an interaction between emotion and congruency: *F(*1,174) = 14.31, *p* < 0.001. Planned t.test comparisons revealed that participants estimations were more accurate in Congruent items for neutral than for emotional content: *t(*175) = -3.23, *p* < 0.01. However, this effect was reversed in Incongruent items, participants relied more on statistical information for emotional content than neutral one: *t(*175) = 2.46, *p* = 0.01. This finding goes against previous results in which they found that emotional content impaired statistical reasoning specifically in Incongruent items [30].

**Table 5:** Results Experiment 2c.

	Neutral		Emotional	
	Congruent	Incongruent	Congruent	Incongruent
Native Language	88.20 (*22*.*51*)	-41.26 (65.73)	77.41 (*42*.*38*)	-24.20 (*76*.*85*)
Foreign Language	88.04 (*20*.*28*)	-50.80 (65.29)	77.20 (*41*.*74*)	-36.73 (*66*.*27*)
Difference	0.06	9.54	0.21	12.53

Subjects’ averaged estimate of the likelihood to have given the correct response broken down by type of content (Neutral or Emotional), congruency, and language. Estimations were reversed when subjects answered against the base-rate (standard deviations in parenthesis).

Language did not have any effect on overall ratings: *F(*1,174) = 1.36, *p* = 0.24, nor it interacted with neither emotion or congruency conditions: *F(*1,174) = 0.41, *p* = 0.52; *F(*1,174) = 1.68, *p* = 0.19, respectively. Replicating Experiment 2b for the neutral items and now as well with emotional content, foreign language did not reduce the use of the representativeness heuristic (see Table 5).

The results of Experiments 2a-2c show no evidence that language context affects people’s tendency to guide their responses according to the representativeness heuristic. Hence, we fail to find evidence that language context alters people’s reliance on stereotypical information when making probability judgments.

## Discussion

We have presented six studies with more than 2000 participants aiming to explore the foreign language effect on decision making. As argued, foreign language context affects people’s choices in decision making situations where emotion plays a key role. Here we assess whether such linguistic context may also alter people’s choices in more emotionally neutral situations that lead to well-known biases: the outcome bias, the conjunction and base-rate neglect fallacies.

Our results show no evidence that these fallacies are affected by language context. In Experiments 1a-1c, people were equally affected by the outcome bias independently from the language context they were set in. In Experiments 2a-2c, people used the representativeness heuristic to the same extent in a foreign and native language context.

These results can be interpreted as indexing the boundaries of the foreign language effect. They are partially consistent with the notion that the foreign language effect is only (or mostly) present in decision making contexts in which people’s choices are driven by an emotional reaction. In such view, foreign language contexts do not promote deliberation per se, but rather they diminish the emotional connotation that a situation elicits, thus changing the outcome of the decision. If this is the case, linguistic contexts (native vs. foreign) should not affect people’s choices in more emotionally-neutral scenarios. We say that our results are only “partially” consistent with this view because we fail to observe an effect of language context when the scenarios the application of the representativeness heuristic included emotional connotations (Experiment 2c). Furthermore, when people were asked about how much regret they felt for their decision, this emotion was not reduced in a foreign language when receiving a negative outcome (Experiment 1c). Thus, one might argue that the foreign language effect is not present even in contexts that elicit an emotional reaction. We do not think that this is the right way to characterize the results. This is because it is unclear whether emotion is the force leading to the outcome bias and of the representativeness heuristic. Hence, even if the actual contexts of those experiments contained some emotional context we cannot be certain that such property modulates the presence of the two effects.

### Sharpening the contexts in which a foreign language effect is present

The interpretation of foreign language effects has been mostly framed around the concepts of increase deliberation or reduced emotionality. However, as the evidence regarding the contexts in which such effect is present (or absent) accumulates a more detailed characterization might be possible. We believe that an interpretation of the pattern of effects in the context of the Stanovich’s taxonomy of biases in decision-making can be useful [31].

Stanovich classifies heuristics and biases in three different types depending on the mechanism that is supposed to be behind those decision-making tendencies. He argues that one type is caused by the Default to the Autonomous Mind, defined as the tendency to accept the automatic response caused by intuition or affective responses without further deliberation. This would be the classical intuitive response characterized by dual-system theories [32]. A second type is due to the Focal Bias, the tendency to take for granted how a given problem is explained. This would be characteristic of framing effects that cause people to attend a situation in a specific way because how it was described. And finally, a third type is the Override Failure, which is people’s incapacity to inhibit an automatic response despite of being aware that another possible answer might be correct. This would be related to situations in which people do realize that there are alternative responses to the situation and can even feel the conflict with the alternatives–in contrast with the Default to the Autonomous Mind (see [29] for this argument in similar base-rate scenarios used in Experiment 2b, and [33] for an updated version of Stanovich’s taxonomy, specifically about the relationship between conflict dection and override failure).

A closer look at the contexts in which foreign language effects have been reported reveals that they all fall in the first two types: The Default to the Autonomous Mind or the Focal Bias classes. For example, decision-making tendencies caused by emotion and affect substitution (e.g. loss aversion), which is the type most foreign language effects can be characterized, can be classified as a Default to the Autonomous Mind. The other scenario that is sensitive to foreign language use is framing effects, which is thought to belong to the Focal Bias typology. Interestingly, all the contexts tested here–outcome bias, conjunction and base-rate fallacies- arguably fit into the third type, the Override Failure. Importantly, we did not study these contexts with the prior intention to use Stanovich’s taxonomy, but rather we believe that this taxonomy can give a preliminary account to why we failed to find an effect of foreign language use. Perhaps, the biases and fallacies that are due to this inability to inhibit responses in the presence of conflicting information cannot be modulated by linguistic contexts. Interestingly, although no significant effect of language was found, one pattern is consistent across experiments: people in a foreign language context were slightly more affected by the outcome bias (Experiments 1a, 1b & for goodness of decisions for Experiment 1c), and the representativeness heuristic (heart item for Experiment 2a, incongruent items for Experiments 2b & 2c). This might be related to the increase in cognitive load when using a foreign language. Also, it goes in line with Stanovich’s taxonomy: a higher cognitive load in a foreign language context might reduce the capability to override the automatic response, hence the trend observed. Further research could explore this and use Stanovich’s taxonomy in a more theoretically driven approach.

The tentative characterization described above raises a more fundamental question: Why does foreign language context affect decision making tendencies driven by the Autonomous Mind and the Focal Bias? Besides explanations based on emotional reactivity, one may consider the reduced mental imagery elicited by a foreign language context as a relevant factor [34]. Mental imagery, the ease with which one creates mental images of a given situation, is argued to be a property of the Default to the Autonomous Mind [31]. Thus, a disruption of the capability to create vivid mental images might modify the intuitive responses elicited by the autonomous mind (similar arguments can be made in the moral domain as well [23]).

A different mechanism might be driving the foreign language effect found in the other typology, the Focal bias. Foreign language processing impairs semantic spreading and language prediction [35,36]. Someone in a foreign language context might be especially focused in getting the broad picture and understanding the main message of the situation. This might prevent people from being affected by how the information is framed. Further research could elucidate to which extent this is the driven mechanism of foreign language effect in framing decisions.

To conclude, we fail to find evidence that foreign language context affects the presence of an outcome bias and the application of representativeness heuristic in people’s choices. This reveals certain boundaries regarding the scope of the foreign language effect on people’s decisions. We tentatively hypothesize that other bias and fallacies that result from the inability to inhibit not emotionally laden automatic responses will not be affected by language context. Foreign language use might make you “piensa twice” [5] but only when an emotional reaction is stopping you from doing so.

## Supporting information

S1 File Materials used in all the experiments in Spanish and English. A file with the scenarios used in the languages in which participants were tested.
(PDF)
