# Supplementary material for: The limits of the foreign language effect on decision-making: The case of the outcome bias and the representativeness heuristic
Source: PLoS One. 2018 Sep 7;13(9):e0203528. doi: 10.1371/journal.pone.0203528 (PMC6128570; doi:10.1371/journal.pone.0203528)
Supplement: S1 File — A file with the scenarios used in the languages in which participants were tested. (PDF) [file pone.0203528.s001.pdf]

Experiments 1a & 1b

Participants read the following scenario (both conditions in Experiment 1a, only one condition in Experiment 1b) in either their native language (Spanish) or foreign language (English).

Spanish:

Un hombre de 55 años tenía una enfermedad de corazón. Tuvo que parar de trabajar por el dolor que le causaba. Le gustaba su trabajo y no quería parar. El dolor también le había afectado otros aspectos de la vida, como viajar y el ocio. Una operación en el corazón disminuiría su dolor e incrementaría su esperanza de vida de 65 años a 70. No obstante, un 8% de las personas que pasan la operación mueren por la propia operación. Su médico decidió realizar la operación. La operación falló y el hombre murió. (o La operación funcionó bien y el hombre se recuperó.)

Evalúa la decisión del doctor de llevar a cabo la operación en una escala del 1 (muy mala) al 7 (excelente).

(incorrecta, una decisión muy mala)	(incorrecta, todas las cosas consideradas)	(incorrecta, pero razonable)	(la decisión y lo contrario eran igual de buenas)	(correcta, pero lo opuesto hubiese sido razonable también)	(correcta, todas las cosas consideradas)	(claramente correcta, una decisión excelente)
1	2	3	4	5	6	7

English:

A 55-year-old man had a heart disease. He had to stop working because of the pain it caused him. He enjoyed his work and did not want to stop. His pain also interfered with other aspects of his life, such as travel and recreation. A heart operation would reduce his pain and increase his life expectancy from age 65 to age 70. However, 8% of the people who have this operation die from the operation itself. His doctor decided to do the operation. The operation failed, and the man died. (or the operation went well, and the man recovered).

Evaluate the doctor's decision to do the operation on a scale of 1 (very bad) to 7 (excellent):

(incorrect, a very bad decision)	(incorrect, all things considered)	(incorrect, but reasonable)	(the decision and its opposite were equally good)	(correct, but the opposite would be reasonable too)	(correct, all things considered)	(clearly correct, an excellent decision)
1	2	3	4	5	6	7

Experiment 1c

Participants read the following scenario in either their native language (Spanish) or foreign language (English).

Spanish:

Imagina que quieres invertir 5,000€ y tienes que elegir entre dos brókeres, Bróker A y Bróker B. Una inversión exitosa significaría incrementar tus 5,000€ un 15% o más en un año. El Bróker A tiene un 43% de probabilidad de éxito, mientras el Bróker B tiene un 54%. ¿Qué Bróker elegirías?

English:

Imagine that you want to invest 5,000€ and you must choose between one of two brokers, Broker A and Broker B. A successful investment would mean to increase your 5,000€ investment by 15% or more within a year. Broker A has a 43% chance of success, while Broker B has a 54% chance. Which Broker would you choose?

Then, participants completed unrelated tasks for a period of 15 minutes, and after that they either given a positive outcome of their decision or a negative one, and asked to answer the following questions on a scale from 1 (not at all) to 7 (very much):

1. *Do you regret your decision?*
2. *Do you think your decision was a good decision?*
3. *Would you have preferred to choose the other option?*
4. *If you had again 5,000€, which broker would you choose now?*

Experiment 2a

Participants read the following two scenarios in either their native language (Spanish) or foreign language (English). The order of presentation was counterbalanced between participants.

Spanish:

Escenario 1

Elisa tiene 31 años, es soltera y muy lista. Elisa tiene el grado de filosofía. Cuando era estudiante, Elisa estaba muy preocupada sobre problemas de discriminación y justicia social, y también participó en protestas antiglobalización.

Ordena los siguientes enunciados según su probabilidad de más a menos probable.

☐ *Elisa trabaja en un banco*

☐ *Elisa es activista del movimiento feminista*

☐ *Elisa trabaja en un banco y es activista del movimiento feminista*

Escenario 2

Se realizó una encuesta de salud a una muestra representativa de hombres adultos en Barcelona que incluía todas las edades y oficios. Marc fue incluido en la muestra. Fue seleccionado al azar de una lista de participantes.

¿Cuál de estos enunciados es más probable? Selecciona uno.

a. *Marc ha sufrido uno o más ataques al corazón*
b. *Marc ha sufrido uno o más ataques al corazón y tiene más de 55 años*

English:

Scenario 1

Elisa is 31 years old, single and very smart. She has a degree in philosophy. When she was a student, Elisa was concerned about issues of discrimination and social justice, and she participated in anti-globalization protests.

Order the following statements according to their probability from most to least probable:

☐ *Elisa works in a bank*

☐ *Elisa is active in the feminist movement*

☐ *Elisa works in a bank and is active in the feminist movement*

Scenario 2

A health questionnaire was carried out in a representative sample of male adults from Barcelona that included all ages and professions. Marc was included in the sample. He was randomly selected from a list of participants.

Which of these statements is the most probable? Select one.

a. *Marc has suffered one or more heart attacks*
b. *Marc has suffered one or more heart attacks and is more than 55 years old*

Experiment 2b

Participants read the following scenarios in either their native language (Spanish) or foreign language (English). The order of presentation was counterbalanced between participants.

Spanish:

Vas a leer tres descripciones de personas que participaron en estudios distintos. En cada caso tienes que leer cuidadosamente la información dada y decidir cuál de las dos opciones es más plausible.

Caso 1 (Incongruent)

100 personas participaron en este estudio. 90% eran abogados y 10% eran ingenieros. Javier fue uno de los participantes del estudio.

Javier tiene 36 años. No está casado y en cierto modo es introvertido. Le gusta pasar su tiempo libre leyendo ciencia ficción y escribiendo programas de ordenador. ¿Qué es lo más probable?

a. *Javier es abogado*
b. *Javier es ingeniero*

Caso 2 (Congruent)

100 personas participaron en este estudio. 90% tenían un tatuaje y 10% no tenían ninguno. José fue uno de los participantes del estudio.

José tiene 29 años. Ha pasado un corto período de tiempo en la cárcel. En este momento lleva 2 años viviendo por su cuenta. Tiene un coche antiguo y escucha música punk. ¿Qué es lo más probable?

a. *José no tiene un tatuaje*
b. *José tiene un tatuaje*

Caso 3 (Neutral)

100 personas participaron en este estudio. 90% tocaban la trompeta y 10% tocaban el saxofón. Toni fue uno de los participantes del estudio

Toni tiene 20 años. Está estudiando en Barcelona y está soltero. Se acaba de comprar un coche de segunda mano con sus ahorros. ¿Qué es lo más probable?

a. *Toni toca la trompeta*
b. *Toni toca el saxofón*

English:

You will read three descriptions of people who participated in different studies. In each case you have to read the information given carefully and decide which of the two options is most plausible.

Case 1 (Incongruent)

100 people participated in this study. 90% were lawyers and 10% were engineers. Jack was one of the participants of the study.

Jack is 36 years old. He is not married and is somewhat introverted. He likes to spend his free time reading science fiction and writing computer programs. What is most likely?

a. *Jack is a lawyer*
b. *Jack is an engineer*

Case 2 (Congruent)

100 people participated in this study. 90% had a tattoo and 10% had none. Jay was one of the participants of the study.

Jay is 29 years old. He has served a short time in prison. He has been living on his own for 2 years now. He has an old car and listens to punk music. What is most likely?

a. Jay has no tattoos

b. Jay has a tattoo

Case 3 (Neutral)

100 people participated in this study. 90% played the trumpet and 10% played the saxophone. Tom was one of the participants of the study.

Tom is 20 years old. He is studying in Barcelona and is single. He just bought a second-hand car with his savings. What is most likely?

a. Tom plays the trumpet

b. Tom plays the saxophone

Experiment 2c

Participants read the following four scenarios in either their native language (Spanish) or foreign language (English). Each participant saw one scenario from each condition (Neutral – Congruent; Neutral – Incongruent; Emotional – Congruent; Emotional – Incongruent). The order of presentation and the specific scenarios was counterbalanced between participants.

Spanish

Scenario Neutral A – Congruent

En una muestra de 1000 personas, 995 son atletas profesionales y 5 son doctores. Erica tiene 22 años. Se pasa la mayor parte de su tiempo entrenando y jugando a baloncesto. ¿Qué es más probable?

a) Erica es una atleta profesional.
b) Erica es doctora.

Scenario Neutral A – Incongruent

En una muestra de 1000 personas, 995 son doctores y 5 son atletas profesionales. Erica tiene 22 años. Se pasa la mayor parte de su tiempo entrenando y jugando a baloncesto. ¿Qué es más probable?

a) Erica es una atleta profesional.
b) Erica es doctora.

Scenario Neutral B – Congruent

En una muestra de 1000 personas, 995 son estudiantes de medicina y 5 son abogados. Juan tiene 25 años. Su sueño es convertirse un día en cirujano cardíaco. ¿Qué es más probable?

a) Juan es abogado.

b) Juan es estudiante de medicina.

Scenario Neutral B – Incongruent

En una muestra de 1000 personas, 995 son abogados y 5 son estudiantes de medicina. Juan tiene 25 años. Su sueño es convertirse un día en cirujano cardíaco. ¿Qué es más probable?

a) Juan es abogado.

b) Juan es estudiante de medicina.

Scenario Emotional C – Congruent

En una muestra de 1000 personas, 995 tienen cáncer y 5 están sanos. Jessica está en la mitad de sus 40 y visita el hospital dos veces a la semana. ¿Qué es más probable?

a) Jessica tiene cáncer.

b) Jessica está sana.

Scenario Emotional C – Incongruent

En una muestra de 1000 personas, 995 están sanas y 5 tienen cáncer. Jessica está en la mitad de sus 40 y visita el hospital dos veces a la semana. ¿Qué es más probable?

a) Jessica tiene cáncer.

b) Jessica está sana.

Scenario Emotional D – Congruent

En una muestra de 1000 personas, 995 están sanos y 5 tienen anorexia. Hugo tiene 25 años, mide 1.82 metros y pesa 95 kg. ¿Qué es más probable?

a) Hugo tiene anorexia.

b) Hugo está sano.

Scenario Emotional D – Incongruent

En una muestra de 1000 personas, 995 tienen anorexia y 5 están sanos. Hugo tiene 25 años, mide 1.82 metros y pesa 95 kg. ¿Qué es más probable?

a) Hugo tiene anorexia.

b) Hugo está sano.

English

Scenario Neutral A – Congruent

In a sample of 1000 people, 995 are professional athletes and 5 are doctors. Erica is 22 years old. She spends most of her time training and playing basketball. What is most likely?

a) Erica is a professional athlete.
b) Erica is a doctor.

Scenario Neutral A – Incongruent

In a sample of 1000 people, 995 are doctors and 5 are professional athletes. Erica is 22 years old. She spends most of her time training and playing basketball. What is most likely?

a) Erica is a professional athlete.
b) Erica is a doctor.

Scenario Neutral B – Congruent

In a sample of 1000 people, 995 are medical students and 5 are lawyers. John is 25 years old. His dream is one day to become a heart surgeon. What is most likely?

a) John is a lawyer
b) John is a medical student.

Scenario Neutral B – Incongruent

In a sample of 1000 people, 995 are lawyers and 5 are medical students. John is 25 years old. His dream is one day to become a heart surgeon. What is most likely?

a) John is a lawyer.
b) John is a medical student.

Scenario Emotional C – Congruent

In a sample of 1000 people, 995 have cancer and 5 are healthy. Jessica is in her mid-40s and visits the hospital twice a week. What is most likely?

a) Jessica has cancer.
b) Jessica is healthy.

Scenario Emotional C – Incongruent

In a sample of 1000 people, 995 are healthy and 5 have cancer. Jessica is in her mid-40s and visits the hospital twice a week. What is most likely?

a) Jessica has cancer.
b) Jessica is healthy.

Scenario Emotional D – Congruent

In a sample of 1000 people, 995 are healthy and 5 have anorexia. Hugh is 25 years old, 1.82 m tall and weighs 95 kg. What is most likely?

a) Hugh has anorexia.
b) Hugh is healthy.

Scenario Emotional D – Incongruent

In a sample of 1000 people, 995 have anorexia and 5 are healthy. Hugh is 25 years old, 1.82 m tall and weighs 95 kg. What is most likely?

a) Hugh has anorexia.
b) Hugh is healthy.
